# Wogonin attenuates the pathogenicity of *Streptococcus pneumoniae* by double‐target inhibition of Pneumolysin and Sortase A

**DOI:** 10.1111/jcmm.17684

**Published:** 2023-02-06

**Authors:** Kuan Gu, Lizhong Ding, Zhongtian Wang, Yingying Sun, Xiaozhou Sun, Wenbo Yang, Haihang Sun, Ye Tian, Zeyu Wang, Liping Sun

**Affiliations:** ^1^ Changchun University of Chinese Medicine Changchun China; ^2^ Affiliated Hospital to Changchun University of Chinese Medicine Jilin China

**Keywords:** anti‐infection, pneumolysin, sortase A, *Streptococcus pneumoniae*, wogonin

## Abstract

*Streptococcus pneumoniae* (*S. pneumoniae*) is a major causative agent of respiratory disease in patients and can cause respiratory distress and other symptoms in severe cases. Pneumolysin (PLY) is a pore‐forming toxin that induces host tissue injury and inflammatory responses. Sortase A (SrtA), a catalytic enzyme that anchors surface‐associated virulence factors, is critical for *S. pneumoniae* virulence. Here, we found that the active ingredient of the Chinese herb *Scutellaria baicalensis*, wogonin, simultaneously inhibited the haemolytic activity of PLY and SrtA activity. Consequently, wogonin decreased PLY‐mediated cell damage and reduced SrtA‐mediated biofilm formation by *S. pneumoniae*. Furthermore, our data indicated that wogonin did not affect PLY expression but directly altered its oligomerization, leading to reduced activity. Furthermore, the analysis of a mouse pneumonia model further revealed that wogonin reduced mortality in mice infected with *S. pneumoniae* laboratory strain D39 and *S. pneumoniae* clinical isolate E1, reduced the number of colony‐forming units in infected mice and decreased the W/D ratio and levels of the inflammatory factors TNF‐α, IL‐6 and IL‐1β in the lungs of infected mice. Thus, wogonin reduces *S. pneumoniae* pathogenicity by inhibiting the dual targets PLY and SrtA, providing a treatment option for *S. pneumoniae* infection*.*

## 1 INTRODUCTION

*Streptococcus pneumoniae* (*S. pneumoniae*) is a common Gram‐positive diplococcus that was first independently isolated in 1881. *S. pneumoniae* infection can cause community‐acquired pneumonia, and is often accompanied by high fever, chills, cough and other symptoms, and in severe cases, even meningitis, bacteraemia and otitis media.^1^ Currently, cephalosporins, penicillin G and quinolones^2^, ^3^ are mainly used to treat *S. pneumoniae* infections. Clinical *S. pneumoniae* strains are increasingly becoming more resistant to these drugs.^4^ Widespread epidemics of *S. pneumoniae* pose a great threat to global public health. Therefore, the search for new drugs or new strategies to treat diseases such as pneumonia caused by *S. pneumoniae* infection has become a hot topic.

In the past, only capsular polysaccharides^5^, ^6^ were considered the main virulence factor of *S. pneumoniae*. Further studies have shown that the virulence factors^7^, ^8^, ^9^, ^10^, ^11^ of *S. pneumoniae* also include hyaluronate lyase (Hyl), pneumolysin (PLY), choline‐binding protein A (CbpA) and sortase A (Sortase A). Among them, pneumolysin, a cytolytic toxin belonging to the cholesterol‐dependent cytolysin (CDC) family,^12^, ^13^ is a major virulence factor in all stages of *S. pneumoniae* infection and consists of 471 amino acids. PLY monomers bind to the target cell membrane and interact with other PLY molecules, and finally, approximately 34–50 monomers form a transmembrane macropore, leading to cell death. Li and colleagues^14^ found that acacetin reduced the pore‐forming activity of PLY and weakened the virulence of *S. pneumoniae*. Letsiou and colleagues^15^ suggested that alveolar epithelial cells release membrane vesicles (MVs) in response to pneumococcal PLY to regulate innate immune responses during lung injury. Ding and colleagues^16^ discovered that the natural compound hederagenin inhibited the activity of *S. pneumoniae* haemolysin.

*Streptococcus pneumoniae* sortase A (SrtA)^17^ is a cysteine transpeptidase whose main role is to cleave between the threonine (T) and glycine (G) residues of the LPXTG motif in the surface protein^18^ and anchor the resulting protein to the cell wall. Sortase A (SrtA) is a group of membrane‐bound transpeptidases that are widely distributed in Gram‐positive bacteria and covalently bind surface proteins to the peptidoglycan of the corresponding cell wall, which play a key role in bacterial survival and pathogenicity. Chang and colleagues reported that SrtA‐deficient mutants (ΔsrtA)^19^ formed fewer biofilms and were less able to immobilize fibronectin, fibrinogen and vitronectin. Song and colleagues found that EGCG^20^ inhibits inflammatory responses in *S. pneumoniae*‐infected mice by inhibiting PLY and SrtA activity. Jianfeng Wang found that a natural compound, quercetin,^21^ a SrtA inhibitor, reduces *S. pneumoniae* virulence by reducing biofilm formation.

Currently, the antivirulence activity of natural compounds is receiving increasing attention. Wogonin is a flavonoid that is widely found in the traditional Chinese medicines *Scutellaria baicalensis Georgi* and *Scutellaria barbata*. According to a recent study,^22^ flavonoids regulate the accumulation of reactive oxygen species and possess anti‐inflammatory and antimicrobial properties. Zheng and colleagues^23^ proposed that wogonin may ameliorate renal inflammation in subjects with diabetic nephropathy by inhibiting the NF‐κB and TGF‐β1/Smad3 signalling pathways. Other researchers have investigated whether wogonin is able to treat acute lung injury,^24^, ^25^ asthma,^26^, ^27^ leukaemia,^28^, ^29^ lung cancers^30^ and other diseases. Nevertheless, the potential effects of wogonin on *S. pneumoniae* have not been reported.

In this article, we found that wogonin is a dual‐target inhibitor of PLY and SrtA in *S. pneumoniae*. Wogonin treatment effectively inhibited PLY activity and alleviated the SrtA‐mediated adhesion of *S. pneumoniae* to host cells, and we further investigated the potential therapeutic effects and mechanisms of wogonin in cells and mouse models.

## 2 MATERIALS AND METHODS

### 2.1 Bacterial culture and reagents

The *S. pneumoniae strain* D39 (NCTC 7466), which was donated by Dr. David E. Briles (Department of Microbiology, University of Alabama at Birmingham), was used throughout the experiment. This study used the *S. pneumoniae* strain E1, which originated from the Laboratory Department of the Affiliated Hospital of Changchun University of Traditional Chinese Medicine and was isolated from a respiratory patient. Wogonin (purity: 98%, m/v) was purchased from Chengdu Herpurify Co., Ltd. (Chengdu, Sichuan, China) and dissolved in 2% dimethyl sulfoxide (DMSO; Sigma–Aldrich, St. Louis, MO, USA).

### 2.2 Haemolysis experiment

Ten microliters of PLY(3 mg/mL, 1:10–1:1000 for activity detection, prepared as previous described) were mixed with wogonin (0, 16, 32, 64 and 128 μg/mL respectively) in 195 μL of PBS, and incubated with 5 μL of sheep blood erythrocytes (purchased from Beijing Solarbio Science & Technology Co., Ltd.) at 37°C for 10 min.^20^, ^31^ Next, the supernatant was removed by centrifugation at 3000 rpm for 5 min, and the absorbance at OD_543 nm_ was measured. The PBS and 1% Triton X‐100 treatment groups were used as negative controls and positive controls respectively. The OD_543 nm_ value of the positive control culture was regarded as 100%, the negative control culture was regarded as 0% and the percent haemolysis of the different concentrations of wogonin treatment was calculated by comparison with the control culture. And we have confirmed that wogonin does not affect the activity of sheep red blood cells.

Haemolysis rate = (OD_543 nm_ values of experimental group – OD_543 nm_ values of Negative control)/(OD_543 nm_ values of Positive control – OD_543 nm_ values of Negative control) × 100%.

Additionally, the supernatants of *S. pneumoniae* D39 and *S. pneumoniae* E1 with an OD_600 nm_ = 0.8 were collected by centrifugation (3000 × *g*) at 4°C for 5 min. And the influence of wogonin on the haemolytic activity of supernatants was examined as described above.

### 2.3 MIC determination

*Streptococcus pneumoniae* D39 or *S. pneumoniae* E1 was incubated in a 37°C incubator until reaching an OD_600 nm_ of approximately 0.5. THB was used to dilute the bacterial broth and dispense it such that the OD_600 nm_ was 0.1. Different concentrations of wogonin or antibiotics (chloramphenicol, amikacin, kanamycin, tetracycline, polymyxin E, gentamycin and vancomycin purchased from Shanghai Yuanye Bio‐Technology Co., Ltd.) were mixed according to the broth microdilution method prior to an incubation for 12 h at 37°C with 5% CO_2_. The MIC indicates the lowest concentration of the tested antibiotics at which the microorganism did not show visible growth.

### 2.4 Determination of *S. pneumoniae* growth

*Streptococcus pneumoniae* D39 or *S. pneumoniae* E1 was inoculated into THY medium at 37°C and grown until reaching an OD_600 nm_ of 0.3. Then, the bacteria were further cultured with different concentrations of wogonin. The OD_600 nm_ was measured every hour for 6 h. Finally, all the data were plotted as a curve.

### 2.5 Analysis of the inhibition of SrtA peptidase activity

Evaluation of the inhibitory effect of wogonin on Sortase A (prepared by Song and colleagues previous described) was performed using the fluorescence resonance energy transfer (FRET) method with reference to the cleavage of peptide substrate (GL Biochem Shanghai, China).^20^, ^31^, ^32^ The reaction buffer was prepared by diluting the SrtA substrate to 10 μM. The SrtA protein concentration was diluted to 5 μM, and the wogonin concentration was diluted to 16, 32, 64 and 128 μg/mL. The final concentrations of wogonin in the sample group were 16, 32, 64 and 128 μg/mL, and the samples were mixed well and incubated at 37°C for 30 min. The fluorescence intensity of the samples was measured with a microplate reader (TECAN, Grodig, Austria) at 0 h. Ten microliters of the substrate solution were added, and the sample was mixed well and incubated for 60 min; the fluorescence intensity of the samples was measured using a microplate reader at 1 h. Wogonin alone did not affect the activity of the absorbance of peptide substrate. SrtA peptidase activity was given by the following equation: [(S – S0)/(C – C0)] × 100, where S and S0 are the fluorescence intensities of the sample group at 1 and 0 h, respectively, and C and C0 are the fluorescence intensities of the positive control group at 1 and 0 h respectively.

### 2.6 Inner membrane permeability test

*Streptococcus pneumoniae* D39 was cultured until reaching the postexponential growth phase and then suspended in PBS buffer (pH = 7.2) at an OD_600 nm_ = 0.5. Then, 150 μL of cells were mixed with different concentrations of wogonin (0, 8–128 μg/mL) in a 96‐well plate and incubated at 37°C for 30 min. Next, the bacterial cells were probed with 3 μM propidium iodide (PI), and the fluorescence intensity was recorded at an excitation wavelength of 535 nm and emission wavelength of 617 nm.

### 2.7 Biofilm formation assay

*Streptococcus pneumoniae* D39 was inoculated into 2 mL of fresh THY medium and incubated overnight at 37°C and with 5% CO_2_. Different concentrations of wogonin (0, 16, 32 and 64 μg/mL) were added once the culture reached logarithmic growth (OD_600 nm_ = 0.4, 1:100), and the culture (500 μL) was transferred to 24‐well plates following an 12 h incubation and washed three times with PBS. One well was supplemented with 400 μL of 0.1% crystal violet staining solution^33^ and incubated for 1 h. Then, the crystal violet was dissolved by adding 200 μL of 33% glacial acetic acid (v/v). And the OD value at 570 nm was measured.

After washing the biofilm three times with PBS, 200 μL of trypsin were added to each well, and the samples were incubated at 37°C for 5 min. Then, 800 μL of sterile water was added to each well, and the mixture was then transferred to a 2 mL centrifuge tube and shaken for 5 min with a microshaker. Ten microliters of the sample were diluted in a 10‐fold gradient, applied to a blood agar plate and incubated at 37°C with 5% CO_2_ for 24 h. The number of plated bacteria was counted.

### 2.8 Western blot assay

*Streptococcus pneumoniae* D39 was cultured to the logarithmic growth phase (OD_600 nm_ = 0.4), mixed with different concentrations of wogonin (0, 16, 32 and 64 μg/mL), and then incubated for 8 h. The bacterial culture supernatant was collected by centrifugation (5000 × *g*, 10 min). Following an incubation with 5× loading buffer at 100°C for 20 min, the proteins in the supernatants were separated on 10% SDS–PAGE gels and transferred to PVDF membranes with a semidry transfer instrument. Then, the membranes were blocked with 5% skim milk powder at room temperature for 2 h. After an incubation with a mouse‐derived anti‐PLY monoclonal antibody (1:1000; Abcam, Cambridge, UK) at room temperature for 2 h, the PVDF membrane was washed three times with TBST, incubated with an HRP‐labelled goat anti‐mouse secondary antibody (1:2000, Proteintech) at room temperature for 1 h and washed three times with TBST. Then, the membranes were placed faced up in a chemiluminescent imaging analyser for fluorescence development.

### 2.9 Oligomerization analysis

The recombinant PLY protein was mixed with different concentrations of wogonin (0, 16, 32 and 64 μg/mL) in a constant temperature water bath at 37°C for 1 h. Then, 5 × β‐mercaptoethanol‐free loading buffer was added to each sample prior to another 10 min of incubation at 55°C. Following separation on 10% SDS–PAGE gels, PLY oligomerization was detected as described above.

### 2.10 Reverse transcription PCR (RT–PCR) analysis

An RT–PCR assay was performed to determine whether the expression of the *ply* gene was affected by wogonin. First, *S. pneumoniae* D39 was cultured until reaching OD_600 nm_ = 0.3 and then different concentrations of wogonin were added and cells were grown to the postexponential growth phase. Next, total RNA was prepared and reverse transcribed into cDNAs using EasyScript One‐Step gDNA Removal and cDNA Synthesis SuperMix (TransGen, Beijing, China). The *S. pneumoniae* 16S rRNA housekeeping gene was chosen as an internal control to quantify the expression level of the *ply* gene.

### 2.11 Invasion assay

A549 cells (human lung epithelial cells, purchased from ATCC, USA) were cultured in complete medium consisting of DMEM supplemented with 10% fetal bovine serum at 37°C with 5% CO_2_ after inoculation in 24‐well plates with 5 × 10^4^ cells per well for an overnight culture. *S. pneumoniae* D39 was inoculated into 2 mL of fresh THY culture medium, incubated overnight at 37°C with 5% CO_2_ in the presence of different concentrations of wogonin, washed three times with PBS (pH 7.4) by centrifugation (5000 × *g*, 10 min) and resuspended in PBS for subsequent analysis.

Next, A549 cells were cocultured with the above *S. pneumoniae* D39 suspension at an MOI of 30. After coculture for 2 h at 37°C with 5% CO_2_, the culture supernatant was extracted from each well. Then, the A549 culture system was flushed three times with PBS (pH 7.4). Next, the A549 cells were treated with 200 μL of 0.25% trypsin (containing 0.02% EDTA) and lysed with 800 μL of 0.02% Triton X‐100. The number of *S. pneumoniae* D39 bacteria was calculated using the serial dilution and plate counting method. *S. pneumoniae* D39‐infected samples incubated without wogonin were used as positive controls, and cells without any treatment were used as negative controls.

### 2.12 LDH release and live/dead cell assay

J774A.1 cells (mouse monocyte macrophage line, ATCC, USA) and human lung epithelial cells were used to determine whether wogonin was potentially toxic to different cells. All cells were seeded into 96‐well plates at 5 × 10^4^ cells/well and incubated at 37°C in CO_2_ incubators overnight. Next, the cells were incubated with various concentrations of wogonin for 6 h at 37°C. Then, the 96‐well plate was centrifuged at 1000 rpm for 10 min to obtain supernatants. The supernatants were collected to confirm the cytotoxicity of wogonin by detecting lactate dehydrogenase (LDH) activity detection (Roche, Mannheim, Germany).

PLY was incubated with wogonin and cocultured with A549 cells at 37°C for 20 min. Following centrifugation (1000 × g, 10 min), 100 μL of supernatant were added to a new 96‐well plate. Then, 100 μL of LDH detection reagent were added. The reaction was conducted for 30 min under low light, and the OD_490 nm_ value of the reaction solution was detected according to the instructions of the cytotoxicity kit. In addition, the cells were treated with 100 μL of live/dead cell staining reagent and observed under a laser confocal microscope. The live cells were stained green, while the dead cells were stained red.

### 2.13 *S. pneumonia*e infection mouse pneumonia model

Female BALB/c mice weighing between 20 and 22 g at 8 weeks were obtained from the Experimental Animal Center of Changchun University of Traditional Chinese Medicine. *S. pneumoniae* D39 was cultured at 37°C in THB to an OD600 nm of 0.4, collected via centrifugation (1000 rpm for 10 min) and washed three times with PBS.

Fifteen mice in each group were slightly anesthetized by inhaling ether and intranasally infected with 20 μL of *S. pneumoniae* D39 (5 × 10^7^ CFUs) in the left nostril. Mice in the uninfected group inhaled an equal volume of sterile PBS. Mice in the *S. pneumoniae* D39‐infected mice were administered wogonin subcutaneously (100 mg/kg) or DMSO every 8 h for 3 days. The infected mice were observed for survival analysis for up to 120 ho. For other analyses, 48 h after infection and administration, the mice were killed, and the lung tissues were removed, fixed with paraformaldehyde, sectioned and stained with haematoxylin–eosin to observe the degree of lung inflammation. The left lung was weighed and then dried at 70°C for 72 h, and the wet/dry ratio was determined. Furthermore, the final serum was centrifuged, and the inflammatory factors were detected using an ELISA kit (Sigma–Aldrich).

### 2.14 Statistical analysis

Experimental data are presented as the means ± SD and were analysed using SPSS 22.0 statistical software (Chicago, IL, USA) as well as GraphPad Prism 8.0.2 software. Independent Student's *t*‐test was adopted to determine statistical significance. Three replicates were analysed per sample, with *p < 0.05 and **p < 0.01.

## 3 RESULTS

### 3.1 Wogonin inhibits the haemolytic activity of PLY

**FIGURE 1:**
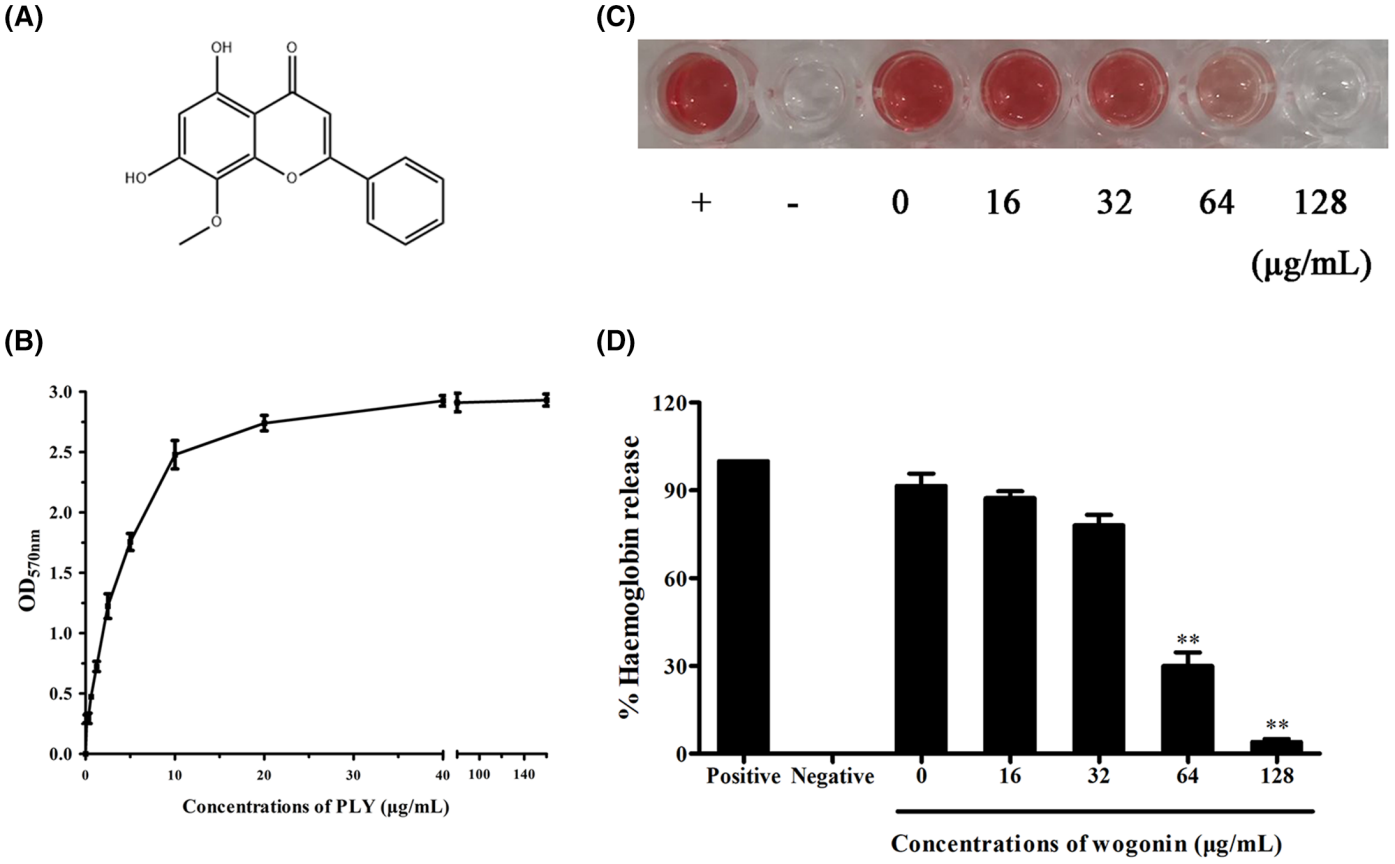
Wogonin inhibits the haemolytic activity of PLY. (A) Chemical structure of wogonin. (B) Determination of PLY activity using a haemolysis assay. The PLY protein was added at concentrations of 0, 10, 20, 30, 40–140 μg/mL to sheep erythrocytes treated with PBS alone, and the haemolysis curve was constructed according to the PLY protein concentration. (C,D) Inhibition of PLY by wogonin. PLY was pretreated with different concentrations (16, 32, 64 or 128 μg/mL) of wogonin, and its haemolytic activity was determined by performing a haemolysis assay. After adding wogonin, the haemolytic activity of PLY decreased significantly. The negative group represents the group containing PBS buffer and erythrocytes without wogonin. The positive group represents the group containing only erythrocytes and water, which could cause 100% haemolysis. Bar graphs indicate the means (*n* = 3), and error bars indicate the standard deviations (SD). **Indicates *p* < 0.01.

PLY is a virulence factor of *S. pneumoniae* that causes erythrocyte haemolysis.^34^ As shown in Figure 1B, when the PLY concentration reached 40 μg/mL, the red blood cells in the culture system were completely lysed, providing an assay of PLY biological activity for use in the next step of screening inhibitors. Wogonin (Figure 1A) is a flavonoid with broad pharmacological activities. After adding different concentrations of wogonin to the haemolytic reaction system, wogonin significantly inhibited the haemolytic activity of PLY in a dose‐dependent manner (Figure 1C,D). The IC_50_ was 52.8 μg/mL. Thus, these results suggest that wogonin is a potent PLY inhibitor.

**FIGURE 2:**
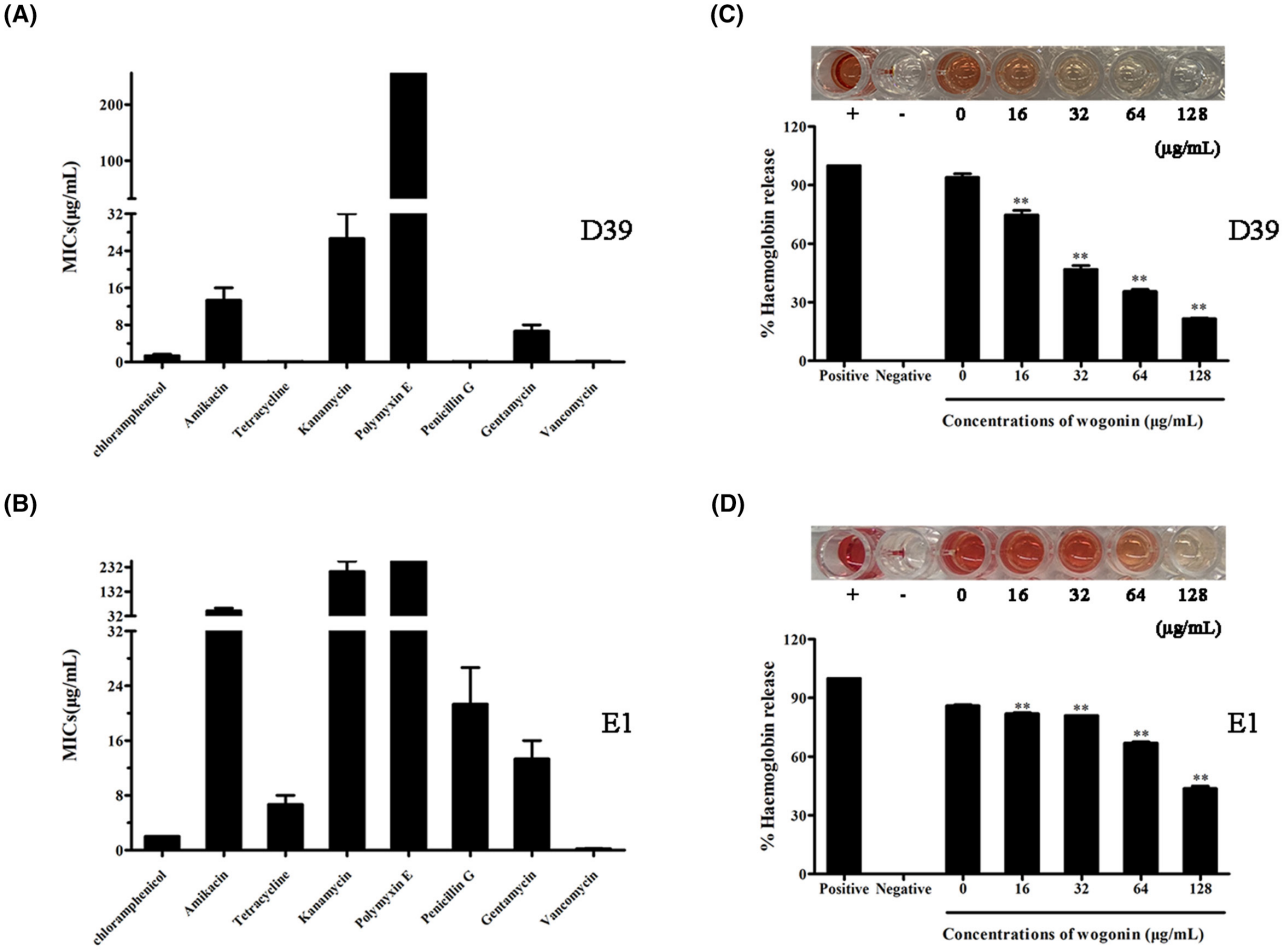
Inhibition of the haemolytic activity of *S. pneumoniae* culture supernatants by wogonin. (A,B) MIC assay of chloramphenicol, amikacin, tetracycline, kanamycin, polymyxin E, penicillin G, gentamycin and vancomycin against *S. pneumoniae* D39 and *S. pneumoniae* E1. (C,D) Coculture with wogonin reduced the haemolytic activity of the *S. pneumoniae* culture supernatant. Each column represents replicates (*n* = 3), and error bars represent standard errors. **Indicates *p* < 0.01.

Furthermore, we tested the resistance of the *S. pneumoniae* laboratory strain D39 and *S. pneumoniae* clinical isolate E1 to different antibiotics. As shown in Figure 2A,B, *S. pneumoniae* D39 exhibited resistance to amikacin, kanamycin and polymyxin (MIC values >8 μg/mL), while *S. pneumoniae* E1 exhibited varying degrees of resistance to all antibiotics tested except vancomycin.

The haemolytic activity of PLY in the supernatants of *S. pneumoniae* D39 (Figure 2C) and *S. pneumoniae* E1 (Figure 2D) was also inhibited in a gradient‐dependent manner after incubation with wogonin. Briefly, our results revealed that wogonin represents an effective inhibitor of PLY in *S. pneumoniae*, whose antibiotic resistance is increasing in the clinical setting.

### 3.2 Wogonin reduces the peptidase activity of SrtA without affecting *S. pneumoniae* growth

**FIGURE 3:**
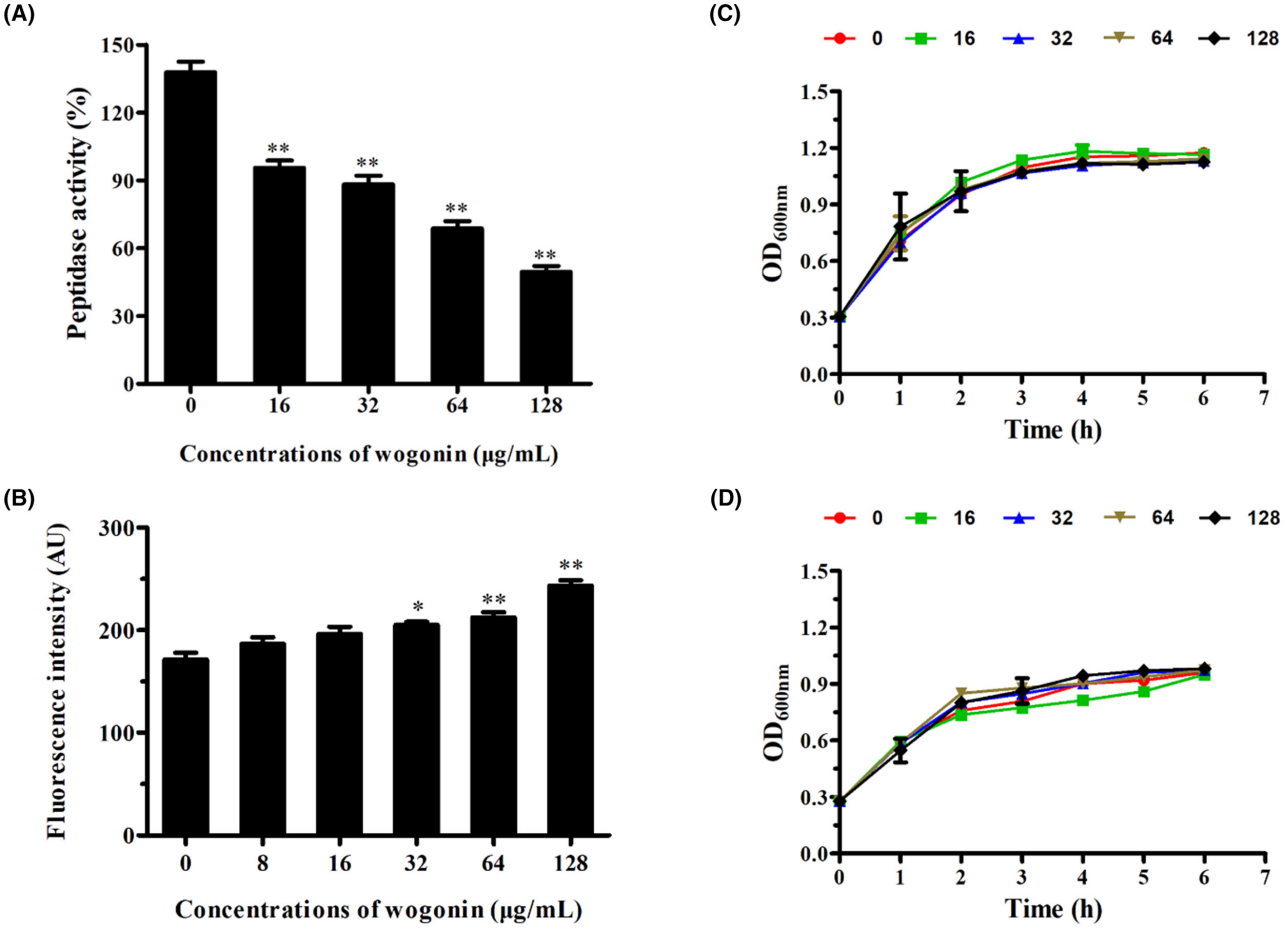
The SrtA inhibitory activity of wogonin. (A) The purified SrtA protein was incubated with different concentrations of wogonin for 30 min. A series of SrtA substrates and fluorescent peptides were added and incubated for 1 h at 37°C. Finally, the fluorescence of the reaction system was measured (the excitation and emission wavelengths were 350 and 520 nm respectively). (B) PI staining was performed to observe the effect of wogonin on the membrane permeability of *S. pneumoniae* D39. Each column represents replicates (*n* = 3), and error bars represent standard errors. *Represents *p* < 0.05 and **indicates *p* < 0.01. (C,D) Coculture of different concentrations of wogonin with *S. pneumoniae* D39 (C) or *S. pneumoniae* E1 (D); the absorbance was measured every hour for 6 h.

The suppressive effect of wogonin on SrtA peptidase activity was defined by performing a FRET assay. Different concentrations of wogonin (0–128 μg/mL) were added to the SrtA activity detection system, and the SrtA peptidase activity decreased in a gradient (Figure 3A). In addition, after the coculture of wogonin and *S. pneumoniae*, the membrane permeability of *S. pneumoniae* was increased with increasing wogonin concentrations (Figure 3B). Furthermore, treatment with wogonin at concentrations that efficiently suppressed PLY activity and SrtA activity resulted in no visible effect on *S. pneumoniae* growth (Figure 3C,D).

**FIGURE 4:**
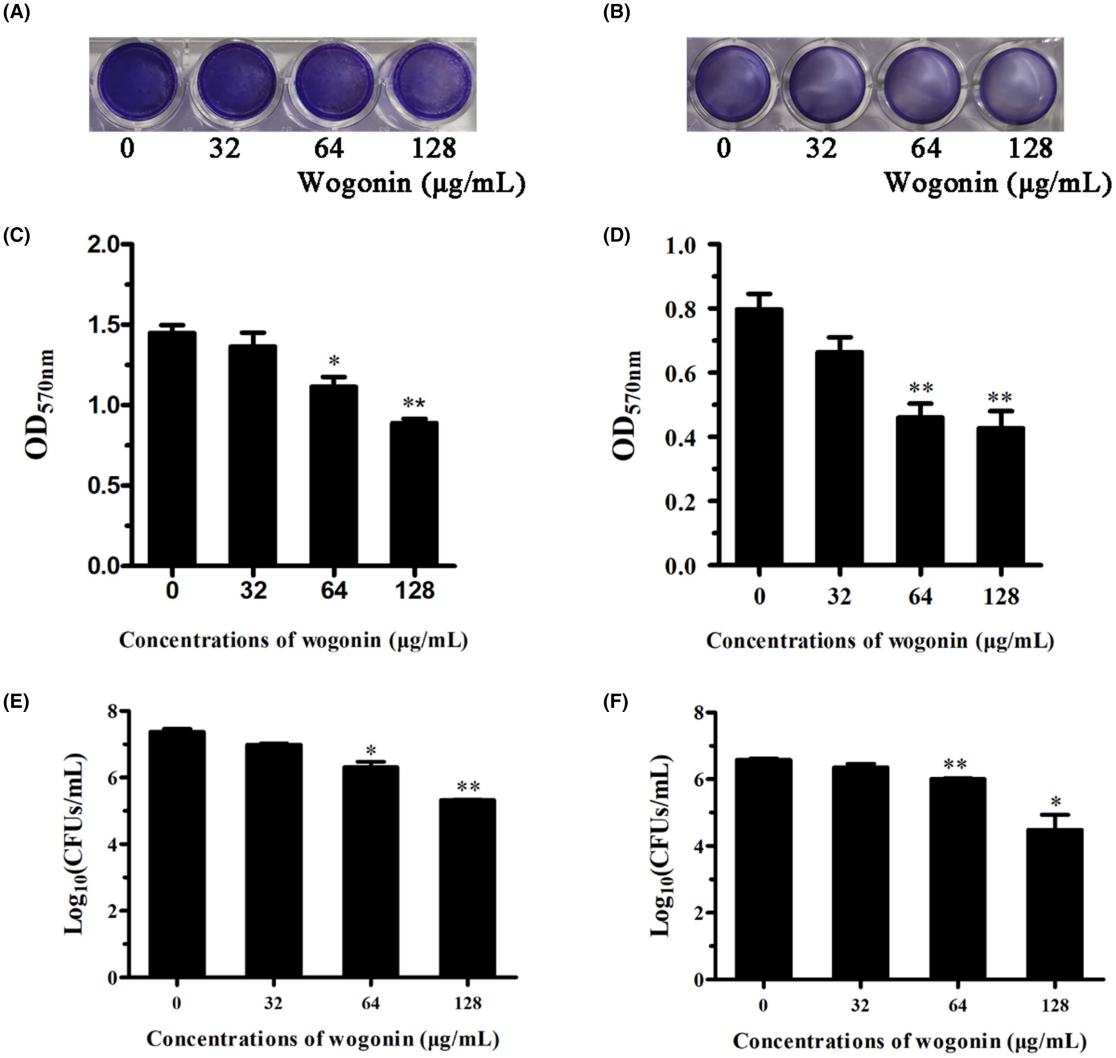
Inhibitory effect of wogonin on *S. pneumoniae* biofilm formation. Photograph of crystal violet‐stained biofilms of *S. pneumoniae* D39 (A) and *S. pneumoniae* E1 (B) that formed in the presence of the indicated concentrations of wogonin. *S. pneumoniae* D39 (C) and *S. pneumoniae* E1 (D) biofilm formation in the presence of the indicated concentrations of wogonin was detected by measuring the amount of bound crystal violet. Number of *S. pneumoniae* D39 (E) and E1 (F) strains in the biofilm (CFUs/mL) formed in the presence of the indicated concentrations of wogonin. Each column represents replicates (*n* = 3), and error bars represent standard errors. *Represents *p* < 0.05 and ** indicates *p* < 0.01.

SrtA inhibitors exert a certain inhibitory effect on the formation of *S. pneumoniae* biofilms.^12^, ^13^ We assessed the effect of wogonin on *S. pneumoniae* D39 and *S. pneumoniae* E1 biofilm formation by performing crystal violet staining and counting biofilm colonies. As the concentration of wogonin increased, the colour of crystal violet‐stained wells became lighter (Figure 4A,B), and the absorbance of the biofilm after acetic acid dissolution also decreased (Figure 4C,D). Simultaneously, the number of bacteria in the biofilm was also significantly reduced (Figure 4E,F). In general, the findings revealed that wogonin is an effective SrtA inhibitor that does not affect *S. pneumoniae* growth.

### 3.3 The dual‐target inhibitory mechanism of wogonin attenuates *S. pneumoniae*‐mediated cell damage

**FIGURE 5:**
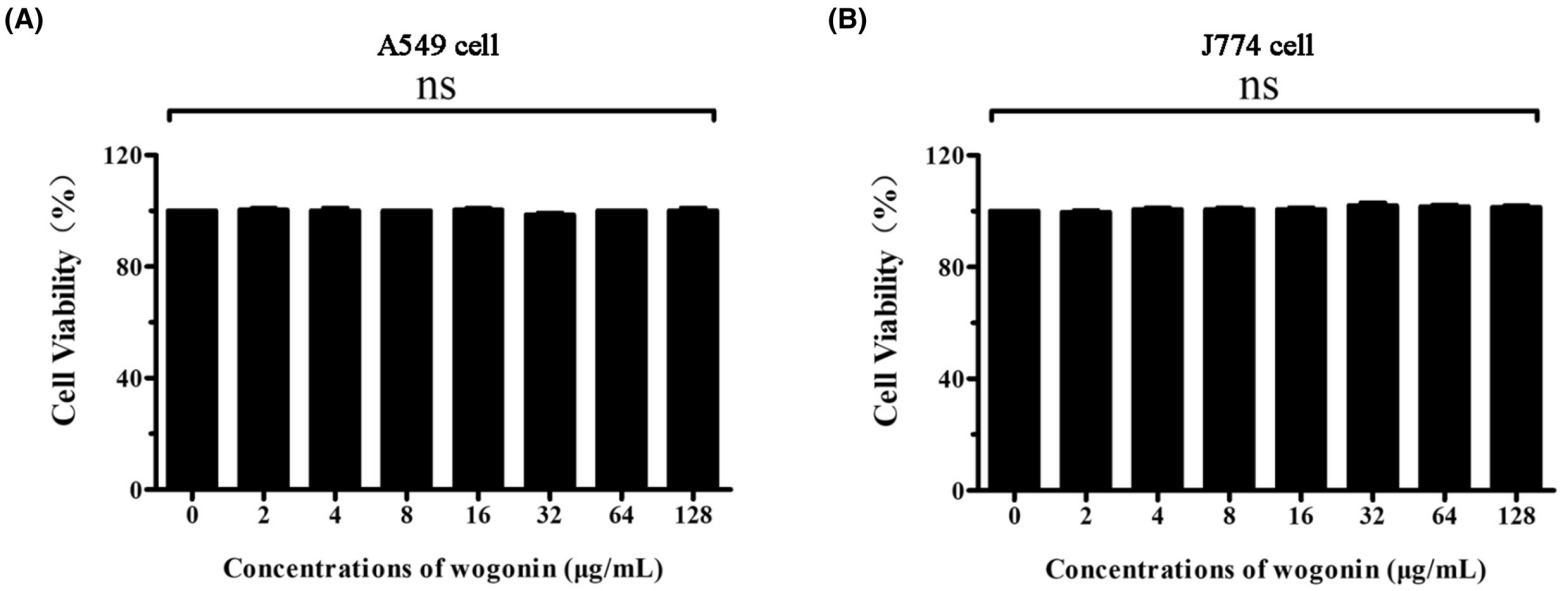
Toxic side effects of wogonin on host cells. A549 (A) and J774 cells (B) were cocultured with different concentrations of wogonin, and LDH release into the supernatant was quantified. Each column represents replicates (*n* = 3), and error bars represent standard errors.

The dual inhibition of PLY activity and SrtA activity by wogonin prompted us to further determine the potential effect of wogonin on *S. pneumoniae* infection in vitro. We added different concentrations of wogonin to A549 cells (Figure 5A) and J774 cells (Figure 5B). When the concentration reached 128 μg/mL, wogonin had little effect on cell viability. Wogonin treatment for 6 h had no obvious cytotoxic effect on A549 and J774 cells.

**FIGURE 6:**
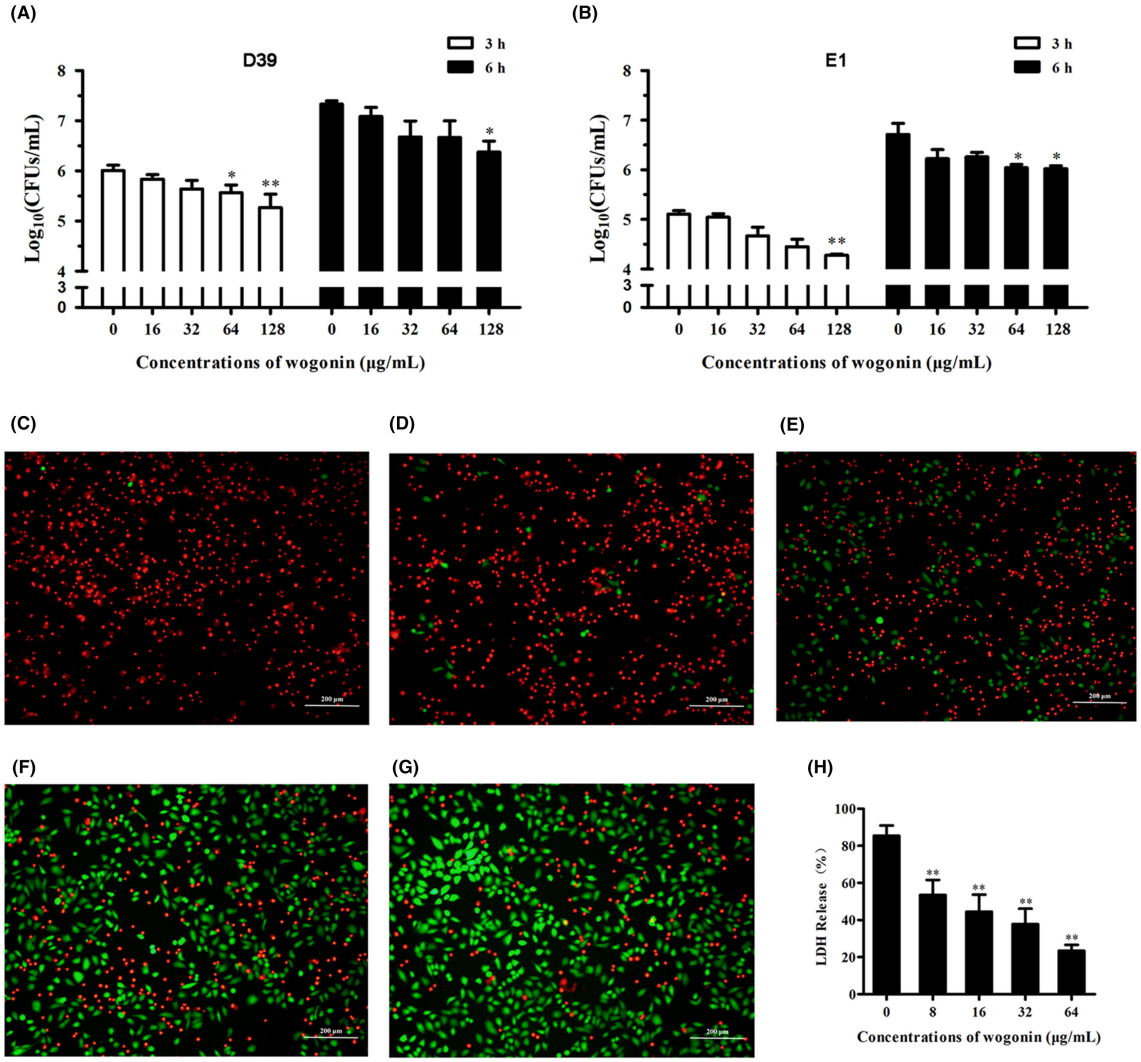
Wogonin inhibits *S. pneumoniae* adhesion and colonization and neutralizes PLY‐mediated injury in A549 cells. (A,B) Wogonin (0, 16, 32, 64 or 128 μg/mL) was added to the *S. pneumoniae* D39 culture system, and the numbers of *S. pneumoniae* D39 and *S. pneumoniae* E1 strains adhering to A549 cells were measured at 3 and 6 h. (C) PLY‐treated A549 cells. Green and red fluorescence read‐outs were imaged using a confocal laser scanning microscope. (D–G) Wogonin was added at concentrations of 8, 16, 32 and 64 μg/mL and incubated with PLY‐treated A549 cells. (H) LDH release by A549 cells in the presence of various concentrations of wogonin. Each column represents replicates (*n* = 3), and error bars represent standard errors. *Represents *p* < 0.05 and **indicates *p* < 0.01.

Then, the effect of wogonin on *S. pneumoniae* adhesion to A549 cells was detected by counting colonies. After counting colonies at 3 h and 6 h, we found that the number of *S. pneumoniae* D39 (Figure 6A) and *S. pneumoniae* E1 (Figure 6B) adhering to A549 cells decreased with wogonin treatment. Therefore, wogonin attenuates the ability of *S. pneumoniae* to adhere to and colonize A549 cells.

PLY not only destroys red blood cells but also directly damages epithelial cells.^35^ Therefore, we added different concentrations of wogonin to the culture system to evaluate whether it exerted a protective effect on A549 cells against PLY. Next, A549 cells were stained with live/dead (green/red staining) reagents. As shown in Figure 6C, almost all A549 cells died after coculture with PLY for 5 h. In contrast, 8 and 16 μg/mL wogonin showed weak protection against cell damage (Figure 6D,E). When wogonin was added at 32 μg/mL (Figure 6F) and 64 μg/mL (Figure 6G), the survival rate of A549 cells was significantly increased in a concentration‐dependent manner. The protective effect of wogonin on A549 cells was further determined by performing a lactate dehydrogenase (LDH) assay. Consistent with the above results, wogonin treatment exerted a similar protective effect on PLY‐mediated cytotoxicity in A549 cells (Figure 6H). Thus, these findings established that PLY and SrtA are the potential targets as wogonin treatment for *S. pneumoniae* infection.

### 3.4 Wogonin reduces PLY oligomerization

**FIGURE 7:**
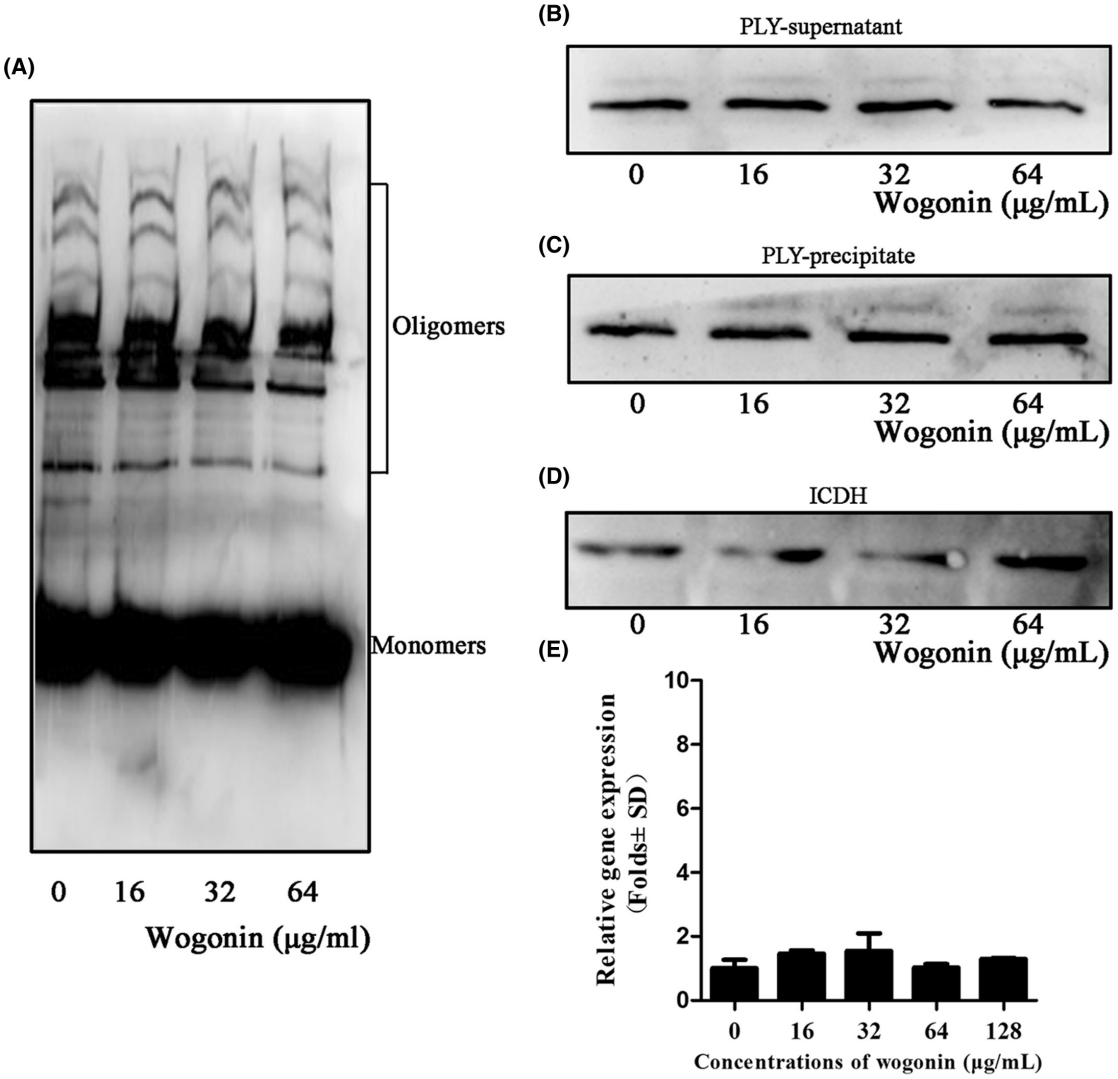
Wogonin inhibits the formation of PLY oligomers without affecting PLY transcription and expression. (A) The effect of wogonin on PLY oligomerization. Purified PLY was incubated with different concentrations of wogonin (0, 16, 32 or 64 μg/mL), and the PLY oligomers were detected by western blotting. (B) Western blot analysis showed that wogonin did not alter the level of PLY in the supernatant. (C) Western blot analysis showed that wogonin did not affect the expression of PLY in precipitates. (D) ICDH was used as an internal reference for the detection of PLY expression. (E) At the mRNA level, wogonin did not affect PLY expression. Each column represents replicates (*n* = 3), and error bars represent standard errors.

PLY is a pore‐forming toxin belonging to the CDC family, whose members bind cholesterol in cell membranes and lyse cells through the oligomerization of soluble monomers to form relatively large pores. Western blot analysis showed that PLY oligomerization was significantly reduced after wogonin incubation in a dose‐dependent manner (Figure 7A). Furthermore, the expression of PLY in the bacterial supernatant and cells was not visibly affected by wogonin treatment (Figure 7B–D). At the transcript level, the addition of wogonin had no effect on the transcription of *ply* the gene encoding PLY (Figure 7E). These discoveries indicated that wogonin suppressed the haemolytic activity of PLY by inhibiting its oligomerization without affecting its transcription and expression.

### 3.5 Wogonin protects mice from *S. pneumoniae* infection

**FIGURE 8:**
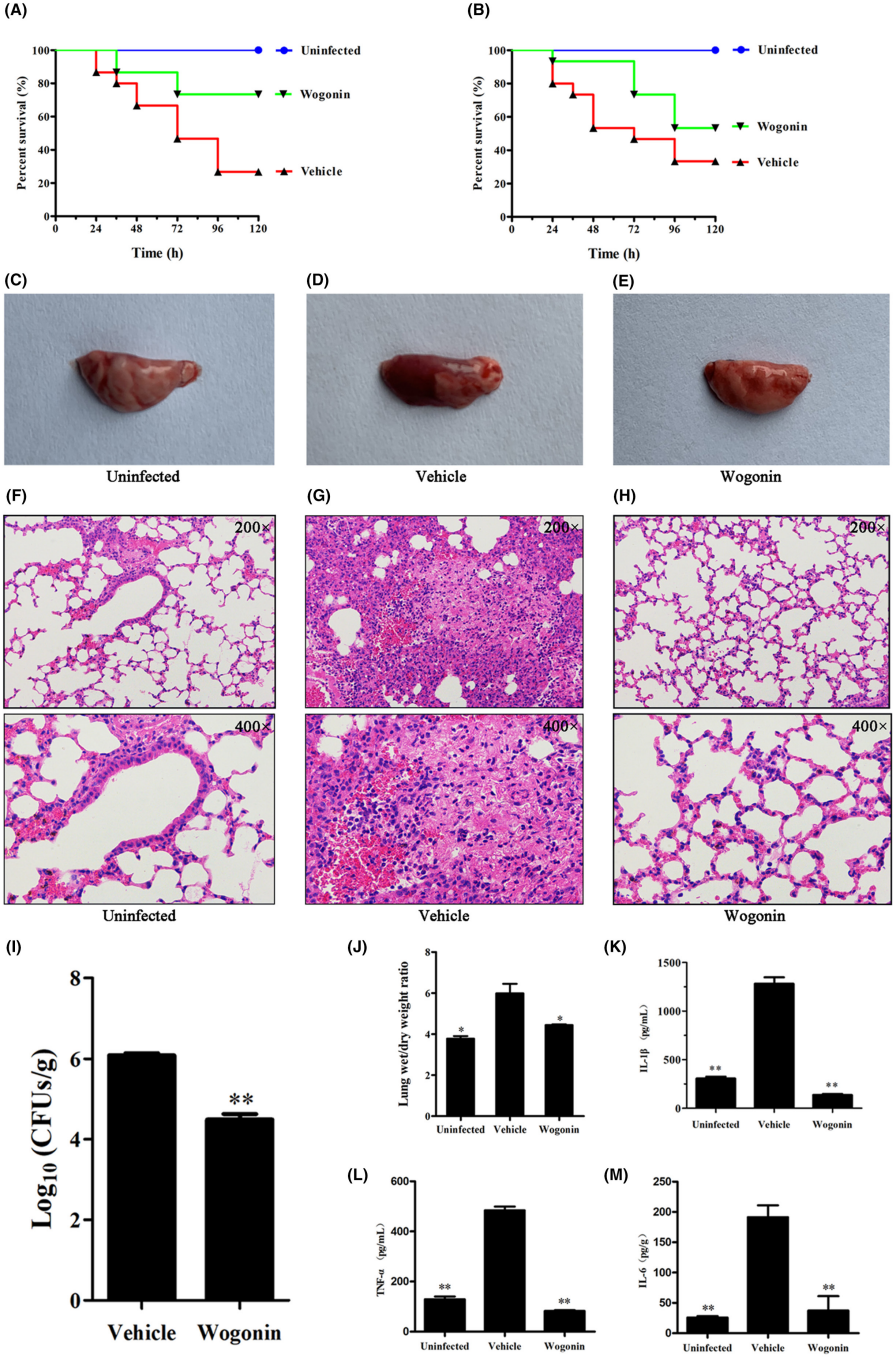
Wogonin reduces the inflammatory response in the mouse lungs. (A) The mortality of mice infected with *S. pneumoniae* D39 for 120 h was examined. (B) The mortality of mice infected with *S. pneumoniae* E1 for a total of 120 h was examined. (C–E) Histopathology of uninfected group, *S. pneumoniae* D39‐infected group and wogonin treatment group, with 15 mice per group. (F–H) Mouse histopathology (200× and 400×). (I) Number of *S. pneumoniae* D39 strains colonized in the lungs. (J) Lung tissue wet/dry ratio. (K–M) The levels of IL‐1β, TNF‐α and IL‐6 in BAL fluid were detected using mouse ELISA kits. Each column represents replicates (*n* = 3), and error bars represent standard errors. *Represents *p* < 0.05 and **indicates *p* < 0.01.

To further validate the protective function of wogonin in vivo, we established the *Streptococcus pneumoniae* D39/E1 infection mouse pneumonia model. The survival of *S. pneumoniae*‐infected mice was observed for 120 h. The 120 h survival rate of *S. pneumoniae* D39‐infected mice was 26.67%, which was increased to 73.33% after wogonin treatment (Figure 8A). Similarly, the 120 h survival rate of *S. pneumoniae* E1‐infected mice was only 33.33%, while the survival rate after wogonin treatment was 53.33% (Figure 8B), suggesting that wogonin improved the survival rate of *S. pneumoniae*‐infected mice. We then examined the extent of lung tissue damage in the target organ. The lungs of the mice in the infected group were darker in colour and obviously congested, while those in the uninfected group were pale pink with no congestion or oedema (Figure 8C,D). The lung tissue damage in the wogonin treatment group was significantly relieved (Figure 8E). As shown in Figure 8F–H, we found that alveolar interstitial oedema, capillary congestion and adhesion were obvious in the infected mice. Additionally, a large number of inflammatory cells were aggregated, producing obvious consolidations; the alveoli ruptured, and blood cells exuded from the alveolar space. After treatment with wogonin, pulmonary inflammation and alveolar oedema were alleviated in mice.

Wogonin reduced the amount of bacterial colonization in the lungs of *S. pneumoniae*‐infected mice (Figure 8I). The left lung wet/dry weight ratio was determined (Figure 8J), and the lung tissue wet/dry ratio of wogonin treatment mice was significantly decreased. In addition, the serum levels of TNF‐α, IL‐6 and IL‐1β (Figure 8K–M) in the wogonin treatment mice were remarkably lower than those in the infected mice. Overall, wogonin exerted an effective therapeutic effect on *S. pneumoniae*‐infected mice.

## 4 DISCUSSION

The ability of *S. pneumoniae* to spread and colonize in a host is a key aspect of pneumococcal population biology and a prerequisite for invasion.^10^, ^36^ PLY is an intracellular protein expressed in *S. pneumoniae*. Only when autolysin A of *S. pneumoniae* is activated and self‐cleavage occurs is PLY released into the extracellular environment in large quantities. Recent research shows that some traditional Chinese medicine extracts and main compounds^14^, ^37^, ^38^ can effectively inhibit the synthesis of the PLY protein. In this experiment, we found that wogonin could significantly decrease the haemolytic activity of PLY. Furthermore, we also found that wogonin inhibited the haemolysis activity of PLY in the culture supernatant. Based on this finding, wogonin is a potent PLY inhibitor.

Furthermore, our results and other studies all found that clinical *Streptococcus pneumoniae* isolate had higher resistance to common antibiotics such as amikacin and kanamycin than laboratory strains (such as *S. pneumoniae* D39).^39^, ^40^ Therefore, we provided a novel strategy to find dual‐target inhibitors of PLY and SrtA to reduce pathogenicity without influencing the growth of *S. pneumoniae*. By western blotting experiments, we proved that wogonin does not affect the transcription and expression of PLY protein but directly affects the oligomerization of PLY, thereby reducing the toxicity of the PLY protein.

*Streptococcus pneumoniae* expresses genes encoding four sortases^41^ (SrtA, SrtB, SrtC and SrtD), but only SrtA is stably expressed in different isolated strains. The *S. pneumoniae* strain D39 used in this study differs from other *S. pneumoniae* strains in that it does not contain the srtB, srtC and srtD genes. Recently, Kharat and colleagues^19^ found that an *S. pneumoniae* SrtA knockout strain exhibited a significantly decreased colonization time and number of colony‐forming units compared with those of the wild type. Moreover, many natural compounds have also been reported to reduce the adhesion and colonization of *S. pneumoniae* in the host. Here, we also further examined whether wogonin abrogated *S. pneumoniae* biofilm formation and reduced the adhesion and colonization of *S. pneumoniae* on cells. Using a FRET assay, we found that wogonin reduced SrtA activity. Moreover, treatment with increasing concentrations of wogonin decreased the intensity of the crystal violet staining of the *S. pneumoniae* D39 and *S. pneumoniae* E1 strains, and the amounts of bacteria in the biofilm^42^, ^43^, ^44^ showed a decreasing trend. Furthermore, wogonin weakened the adhesion and colonization of *S. pneumoniae* D39 and *S. pneumoniae* E1 on A549 cells, consistent with the previous inference. This finding indicates that wogonin is a dual‐target inhibitor of PLY and SrtA.

In this study, we also found that wogonin improved the survival rate of PLY‐treated A549 cells, with little toxicity to the cells. A similar finding was obtained in vivo. Wogonin increased the survival rate of mice infected with *S. pneumoniae* D39. The examination of TNF‐α, IL‐6 and Il‐1β levels indicated that the inflammatory response was alleviated after wogonin treatment, and the number of colony‐forming units of the strains in lung tissue was also reduced. The pathological analysis of the tissue showed that the pathological reaction was alleviated after wogonin treatment. Thus, wogonin treatment exerted a protective effect on *S. pneumoniae* virulence both in vivo and in vitro. In summary, our study lays the foundation for research on wogonin as a new anti‐*S. pneumoniae* drug that simultaneously targets PLY and SrtA.

## AUTHOR CONTRIBUTIONS

**Kuan Gu:** Investigation (equal); methodology (equal); project administration (equal); validation (equal); visualization (equal); writing – original draft (equal); writing – review and editing (equal). **Lizhong Ding:** Data curation (equal); formal analysis (equal). **Zhongtian Wang:** Data curation (equal); formal analysis (equal). **Yingying Sun:** Methodology (equal); project administration (equal). **Xiaozhou Sun:** Data curation (equal); formal analysis (equal). **Wenbo Yang:** Formal analysis (equal); funding acquisition (equal). **Haihang Sun:** Data curation (equal); investigation (equal). **Ye Tian:** Data curation (equal); formal analysis (equal). **Zeyu Wang:** Conceptualization (equal); data curation (equal); formal analysis (equal); funding acquisition (equal); resources (equal); software (equal); supervision (equal); validation (equal). **Liping Sun:** Data curation (equal); formal analysis (equal); funding acquisition (supporting); supervision (equal); validation (equal); visualization (equal).

## FUNDING INFORMATION

This work was supported by the National Key Research and Development Programme of China (2017YFC1703202), the Jilin Provincial Clinical Research Center of Traditional Chinese Medicine and Pediatrics (20200603008SF) and the Jilin Province Chinese Medicine Science and Technology Project (2020J069).

## CONFLICT OF INTEREST STATEMENT

The authors have no competing financial interests to declare.

## Data Availability

The data that support the findings of this study are available from the corresponding author upon reasonable request.
